# Children with Intestinal Failure are at Risk for Psychopathology and Trauma

**DOI:** 10.1097/MPG.0000000000003939

**Published:** 2023-09-08

**Authors:** Lotte E. Vlug, Jeroen S. Legerstee, Merit M. Tabbers, Aysenur Demirok, Merel W. Verloop, Lotte Bosman, Edmond H.H.M. Rings, René M.H. Wijnen, Marjolein Spoel, Barbara A.E. de Koning

**Affiliations:** From the * Department of Pediatrics, Division of Gastroenterology, Erasmus MC Sophia Children’s Hospital, University Medical Center Rotterdam, Rotterdam, The Netherlands; the † Department of Child and Adolescent Psychiatry/Psychology, Erasmus MC Sophia Children’s Hospital, University Medical Center Rotterdam, Rotterdam, The Netherlands; the ‡ Department of Pediatrics, Division of Gastroenterology, AMC Emma Children’s Hospital, Amsterdam University Medical Center, Amsterdam, The Netherlands; the § Department of Pediatrics, Division of Gastroenterology, Willem Alexander Children’s Hospital, Leiden University Medical Center, Leiden, The Netherlands; the ∥ Department of Pediatric Surgery, Erasmus MC Sophia Children’s Hospital, University Medical Center Rotterdam, Rotterdam, The Netherlands; the ¶ Division of Gastroenterology, Hepatology and Nutrition, the Hospital for Sick Children, Toronto, Canada; the Department of Child and Adolescent Psychiatry/Psychology, Erasmus MC Sophia Children’s Hospital, University Medical Center Rotterdam, Rotterdam, The Netherlands; the Department of Child and Adolescent Psychiatry/Psychology, Erasmus MC Sophia Children’s Hospital, University Medical Center Rotterdam, Rotterdam, The Netherlands; From the Department of Pediatrics, Division of Gastroenterology, Erasmus MC Sophia Children’s Hospital, University Medical Center Rotterdam, Rotterdam, The Netherlands; From the Department of Pediatrics, Division of Gastroenterology, Erasmus MC Sophia Children’s Hospital, University Medical Center Rotterdam, Rotterdam, The Netherlands; the Division of Gastroenterology, Hepatology and Nutrition, the Hospital for Sick Children, Toronto, Canada; the Department of Nutritional Sciences, University of Toronto, Toronto, Canada.

**Keywords:** behavioral, emotional, mental health, parenteral nutrition, short bowel syndrome

## Abstract

**Objectives:**

The objective of this study is to assess the psychopathology and medical traumatic stress in children with intestinal failure (IF) and identify associated risk factors.

**Methods:**

Two-center study, performed from September 2019 until April 2022 (partly during COVID-19 pandemic), including children (1.5–17 years) with IF, dependent on parenteral nutrition (PN) or weaned off PN, treated by a multidisciplinary IF-team. Psychopathology in children was evaluated with a semi-structured interview assessing psychiatric classifications and validated questionnaires assessing emotional (internalizing) and behavioral (externalizing) problems. Medical traumatic stress was assessed with a validated questionnaire. Problem scores were compared with normative data. Associations between clinical characteristics and outcomes were analyzed with linear regression analyses.

**Results:**

Forty-one (of 111 eligible) children were included [median age 8.9 years (interquartile range, IQR 5.5–11.8), 54% female, 73% born preterm]. Median PN-duration was 17.3 months (IQR 6.9–54.0); 17 children (41%) were still PN-dependent. One third of the children met criteria for at least 1 psychiatric classification (compared with 14% in age-matched general population). Anxiety disorders and attention deficit hyperactivity disorder were most common. In school-aged children (n = 29, 6–17 years), significantly increased emotional problems were consistently reported by children (*P* = 0.011), parents (*P* < 0.001), and teachers (*P* = 0.004). In preschool children (n = 12, 1.5–5 years), no significant differences with normative data were found. Subclinical or clinical emotional problems were reported in 19 children (46%). Medical traumatic stress was present in 14%, and 22% of children had received psychological help for trauma before. Lower gastrointestinal related quality of life was associated with more emotional problems, but not PN-duration.

**Conclusions:**

Children with IF, particularly school-aged children, are at risk for psychological problems which is reflected by the high rate of received psychotherapy and the high rate of emotional problems and psychiatric classifications.

**What Is Known**

- Treatment of children with intestinal failure (IF) in multidisciplinary teams has led to improved survival and decreased morbidity.
- Many children with IF have preexisting neurodevelopmental impairment when discharged home on parenteral nutrition, but psychosocial consequences of IF are less known

**What Is New**

- Anxiety disorders (19%), attention deficit hyperactivity disorder (11%), and medical traumatic stress (14%) are common in children with conditions that have resulted in IF and are present in children even after enteral autonomy has been gained.
- School-aged children and those with low gastrointestinal related quality of life are prone to have emotional problems.

Children with intestinal failure (IF), whose gut cannot absorb sufficient nutrients needed for growth, depend on parenteral nutrition (PN) given at home through a central venous line (1). Causes of IF predominantly occur early after birth, but also at older age. Underlying intestinal pathologies are short bowel syndrome [mostly in prematurely born infants after resection for necrotizing enterocolitis (NEC)], motility disorders, and enteropathies (1). IF and PN are associated with frequent complications, including osteoporosis, liver fibrosis, central line infections, and thrombosis leading to frequent hospital visits and admissions (1,2). Treatment in multidisciplinary IF teams has led to improved survival rates and decreased morbidity (3). As the IF population grows older, new challenges concerning long-term neurodevelopmental outcomes and psychosocial functioning have gained attention. Low gestational age, long hospitalization, and multiple surgical procedures are associated with impaired neurodevelopment in IF (4). There are few published studies concerning the psychological impact of home PN on children with IF and their families (5–9). Anxiety and attention problems in children were seen, and parents experienced distress and depressive symptoms. In a recently published study concerning children with IF, NEC diagnosis, immigrant status, and not living with biological parents were associated with psychological outcomes (9). Children with IF experience invasive medical procedures and have often been admitted to the intensive care unit, which can cause post-traumatic symptoms (10). Medical traumatic stress was seen in children with injury, critical, and chronic illnesses (11–13), but has not been researched yet in pediatric IF. In clinical practice, we see that children with IF experience school problems, but also emotional and behavioral problems. Moreover, mental health problems in childhood and adolescence are thought to be predictors of adverse economic, social, and health outcomes in young adulthood (14). It is important to evaluate whether psychological problems are over represented in the pediatric IF population and to evaluate the type of problems. Early assessment and intervention for these problems can prevent psychosocial problems later in life.

We aimed to (1) assess psychopathology (ie, psychiatric classifications and emotional and behavioral problems), (2) assess medical traumatic stress, and (3) identify risk factors for emotional and behavioral problems and medical traumatic stress, in children with IF with current or past PN-dependency.

## METHODS

### Study Design and Subjects

A cohort of children with IF was asked to participate in the present exploratory two-center cross-sectional study. The study took place from September 2019 until April 2022 (partly during COVID-19 pandemic). Inclusion criteria were as follows: (1) diagnosis of IF, (2) either currently receiving home PN or having a history of PN-dependence for >60 days (ie weaned off PN), (3) known at the multidisciplinary IF team from the Erasmus MC Sophia Children’s Hospital (Rotterdam, The Netherlands) or Amsterdam UMC Emma Children’s Hospital (Amsterdam, The Netherlands), and (4) 1.5–17 years old. This age group was chosen because emotional and behavioral problems can be assessed in children aged 1.5 years and above; and from 18 years onwards, patients in The Netherlands are considered adults.

The study was in line with the principles of the Declaration of Helsinki, and was approved by the research ethical committee of the Erasmus Medical Center (MEC 2019-098, Dutch Trial Register NL8964, https://trialsearch.who.int/Trial2.aspx?TrialID=NL8964). Written informed consent was obtained from all participants’ caregivers and from participants above 12 years old.

### Data Collection

Demographic and clinical data were retrieved from the medical charts. Participating children and their primary caregivers (one of their parents) underwent a semi-structured telephonic interview with a psychologist to assess psychiatric classifications, and received online questionnaires concerning emotional/behavioral functioning, medical traumatic stress, and gastrointestinal (GI) complaints. We also asked parents with which previous psychological conditions children were diagnosed and what psychological help they received currently or in the past.

#### Psychopathology

Psychopathology is represented by psychiatric classifications and emotional and behavioral problems.

##### Interview: Psychiatric Classifications

Psychiatric classifications in the child were assessed with the translated and linguistically validated Dutch version of the Mini International Neuropsychiatric Interview Kid Screen (MINI-KID). The MINI-KID is a semi-structured interview, carried out by trained personnel (psychologist), used for determining psychiatric classifications according to the Diagnostic and Statistical Manual of Mental Disorders (DSM-) IV diagnoses (no version of DSM-V available) in children and adolescents aged 6–17 years (15). Both child/adolescent and the primary caregiver were present during the interview with the psychologist. The MINI-KID generates reliable and valid psychiatric classifications (16). A more detailed description of the MINI-KID is provided in File S1, Supplemental Digital Content 1, http://links.lww.com/MPG/D305.

##### Questionnaire: Emotional and Behavioral Problems

Emotional and behavioral functioning in children was assessed with the Dutch versions of the Youth Self Report (YSR, 11–17 years), the Child Behavior Checklist (CBCL, 1.5–5 years and 6–17 years) as parent proxy-report, and the Teacher’s Report Form (TRF, 1.5–5 years and 6–17 years) as daycare staff/teacher proxy-report. The YSR, CBCL, and TRF have shown good reliability and validity (17). These questionnaires are used for quantifying internalizing problems (emotional functioning, ie, problems that are present mainly within the child), externalizing problems (behavioral functioning, ie, problems with regard to conflicts with others and to conflicts in the expectation that others have of the child), and total problems (ie, internalizing and externalizing problems together) (18–20). Higher scores implicate more psychopathology. Scores can be classified into normal, subclinical (ie, at risk of becoming clinical problems, requiring attention), and clinical (ie, requiring treatment) problems. In addition to the composite T-scores, problems can be categorized in syndrome scales, namely: emotionally reactive, anxious/depressed, withdrawn/depressed, somatic complaints, sleeping problems, social problems, attention problems, thought problems, rule-breaking behavior, and aggressive behavior. More details are provided in File S1, Supplemental Digital Content 1, http://links.lww.com/MPG/D305.

#### Medical Traumatic Stress

##### Questionnaire: Medical Traumatic Stress

Medical traumatic stress in children was assessed with the validated Dutch Children’s Responses to Trauma Inventory (CRTI) (21,22). It has a child self-report (8–17 years) and a parent-proxy report (4–17 years). The CRTI assesses how a child feels regarding a traumatic event. It includes 3 symptom clusters of post-traumatic stress disorder: intrusion, avoidance, and arousal. Parents and children were instructed to think about hospital visits/admissions and surgeries when filling out the questionnaire. Higher scores indicate more medical traumatic stress. Children with scores above the 60th percentile and/or fulfilling 2 or 3 symptom criteria were considered to have elevated post-traumatic stress disorder symptoms. This is the cut-off for above-average scores according to the manual of the CRTI (File S1, Supplemental Digital Content 1, http://links.lww.com/MPG/D305) (21,23).

### Statistical Analysis

Descriptives are summarized as median with interquartile range (IQR) for continuous data, or as an amount (n) with percentage (%) for categorical data. Chi-square tests (for categorical data), Mann-Whitney *U* tests (for not-normally distributed continuous data), and independent *t* tests (for normally distributed continuous data) were used to compare characteristics, emotional and behavioral functioning, and medical traumatic stress between the group of children still receiving PN and the group of children weaned off PN. These groups were compared to evaluate whether PN itself or other more generic disease factors influenced psychosocial functioning and if problems resolve once weaned off PN. One-sample *t* tests (for normally distributed continuous data) and 1-sample Wilcoxon signed rank tests (for not-normally distributed continuous data) were used to compare emotional and behavioral problems and medical traumatic stress with normative data (24). Binomial tests were performed to compare the proportion of children reporting subclinical or clinical emotional and behavioral problems with the general population (17%) (19,20).

We used PN-dependency duration and GI related quality of life [measured with the Pediatric Quality of Life inventory – Gastrointestinal Symptoms Module total score (File S1, Supplemental Digital Content 1, http://links.lww.com/MPG/D305)] as proxies of IF severity (25,26). We performed explorative univariable and multivariable linear regression analyses to assess associations of PN-dependency duration, GI-related quality of life, and other clinical characteristics (sex, gestational age, age at time of assessment, duration of hospital stay, number of surgeries) with internalizing/externalizing problems and medical traumatic stress. This was done for the parent-proxy reports, since these encompass all children. Assumptions for multiple linear regression analyses were met (ie, linear relationship of independent and dependent variables, normality of residuals of regression, no multicollinearity and homoscedasticity). Predictors with *P* < 0.4 were then combined in multivariable linear regression analyses with a maximum of 1 predictor added for every n = 10 children. We carried out multivariable analysis to see if possible associations found in univariable analyses were confounded by other variables. More details on statistics are provided in File S2, Supplemental Digital Content 2, http://links.lww.com/MPG/D306.

A 2-tailed *P* value of <0.05 was considered to indicate statistical significance. Due to the exploratory nature of the study, we did not correct for multiple testing. Data analyses were performed using the Statistical Package for the Social Sciences, Version 28.0 (IBM SPSS Statistics for Windows, Armonk, NY).

## RESULTS

### Patient Characteristics

Of 71 eligible children in Rotterdam, 35 agreed to participate in the study (participation rate of 49%). Of 40 eligible children in Amsterdam, 6 agreed to participate (participation rate of 15%). Demographic and IF-related characteristics are presented in Table 1. At the time of assessment, included children had a median age of 8.9 years (IQR 5.5–11.8 years). Seventeen of the included children were receiving home PN for a median of 6.8 years (IQR 3.4–10.7 years), and 24 children were weaned off PN for a median of 7.7 years (IQR 4.9–9.4 years).

**TABLE 1.** Patient characteristics

	Total cohort	Weaned off PN at time of assessment	Receiving PN at time of assessment	*P* value*
	N = 41	N = 24	N = 17	
	n (%) or median [IQR]	n (%) or median [IQR]	n (%) or median [IQR]	
Age at time of assessment, y	8.9 [5.5–11.8]	8.7 [5.5–11.4]	9.3 [5.5–13.3]	0.543
Sex, female	22 (54)	16 (67)	6 (35)	**0.047**
Gestational age, wk (n = 39)	34.0 [28.6–36.7]	31.8 [26.6–34.7]	36.3 [33.7–38.6]	**0.008**
Term (gestational age ≥37 wk)	9 (23)	3 (13)	6 (40)	**0.014**
Moderate/late preterm (gestational age 32–37 wk)	14 (36)	8 (33)	6 (40)	
Very preterm (gestational age 28–32 wk)	7 (18)	5 (21)	2 (13)	
Extremely preterm (gestational age <28 wk)	9 (23)	8 (33)	1 (7)	
IF diagnosed during infancy	37 (90)	24 (100)	13 (76)	**0.024**
Underlying disease				**0.010**
Gastroschisis	4 (10)	4 (17)	0 (0)	
Intestinal atresia	5 (12)	3 (13)	2 (12)	
Necrotizing enterocolitis	14 (34)	11 (46)	3 (18)	
Midgut volvulus	5 (12)	3 (12)	2 (12)	
Intestinal pseudo-obstruction syndrome	5 (12)	0 (0)	5 (29)	
Other	8 (20)	3 (12)	5 (29)	
Suspected or confirmed genetic defect/syndrome	3 (7)	1 (4)	2 (12)	0.560
Number of surgeries under general anesthesia	7 [4–12]	5 [3–9]	13 [5–21]	**0.012**
Total duration of hospital stays, wk	25.0 [14.9–32.0]	24.6 [17.6–31.3]	27.9 [13.7–61.4]	0.931
Total PN-dependency duration, mo†	17.3 [6.9–54.0]	8.1 [5.1–15.4]	81.2 [40.7–128.5]	**<0.001**
Maternal age at child’s birth, y (n = 37)	29.0 [26.0–34.0]	30.5 [27.3–34.8]	28.0 [25.0–30.0]	0.071
Single parent household	4 (10)	4 (17)	0 (0)	0.133
Primary caregiver, male	6 (15)	2 (8)	4 (24)	0.212
Attending special education	9 (22)	3 (13)	6 (35)	0.128

*P* values in bold represent significant differences between the 2 groups.

IF = intestinal failure; IQR = interquartile range; PN = parenteral nutrition.

* Comparison of children weaned off PN at time of assessment with children receiving PN at time of assessment. Tested with Mann-Whitney *U* tests.

† Calculated with Kaplan-Meier survival curve to take into account that patients were still PN-dependent at the time of assessment.

Twenty-three children (56%) had previously reached out for psychological help (77% of children receiving home PN vs 48% of children weaned off PN, *P* = 0.070). Thirteen children (32%) actually received psychological care prior to or during participation in the current study (53% of children receiving PN vs 20% of children weaned off PN, *P* = 0.036). Children received psychological care in the university medical center that they were cared for by the multidisciplinary IF team too. When parents/caregivers had a request for help mentioned to the IF team, they were referred to a psychologist. Most of the problems they were facing and received psychological help for were behavioral problems, traumatic complaints, anxiety problems, and feeding/eating difficulties.

### Psychopathology

#### Interview: Psychiatric Classifications

The MINI-KID interview was assessed in 27 of 29 eligible children [6–17 years old; median age 10.3 years (IQR 8.5–13.0 years)]. Nine children (33%) met DSM-IV criteria for at least 1 current classification, of which 7 children met criteria for multiple psychiatric classifications. Two children (7%) fulfilled criteria for a depressive episode in the past, 6 children (22%) for anxiety disorders [5 (19%) for specific phobia, 3 (11%) for social phobia, 1 (4%) for panic disorder, and 1 (4%) for agoraphobia], 3 (11%) for attention deficit hyperactivity disorder (ADHD) of the inattentive type, 1 child (4%) for an adjustment disorder of the combined type, and 1 child (4%) for autism spectrum disorder. None met criteria for suicidality, bipolar disorders, obsessive compulsive disorders, post-traumatic stress disorders, alcohol abuse, substance abuse, tic disorders, disruptive disorders, psychotic disorders, or eating disorders.

#### Questionnaires: Emotional and Behavioral Problems

##### YSR (Self-Reported Problems)

**FIGURE 1.**
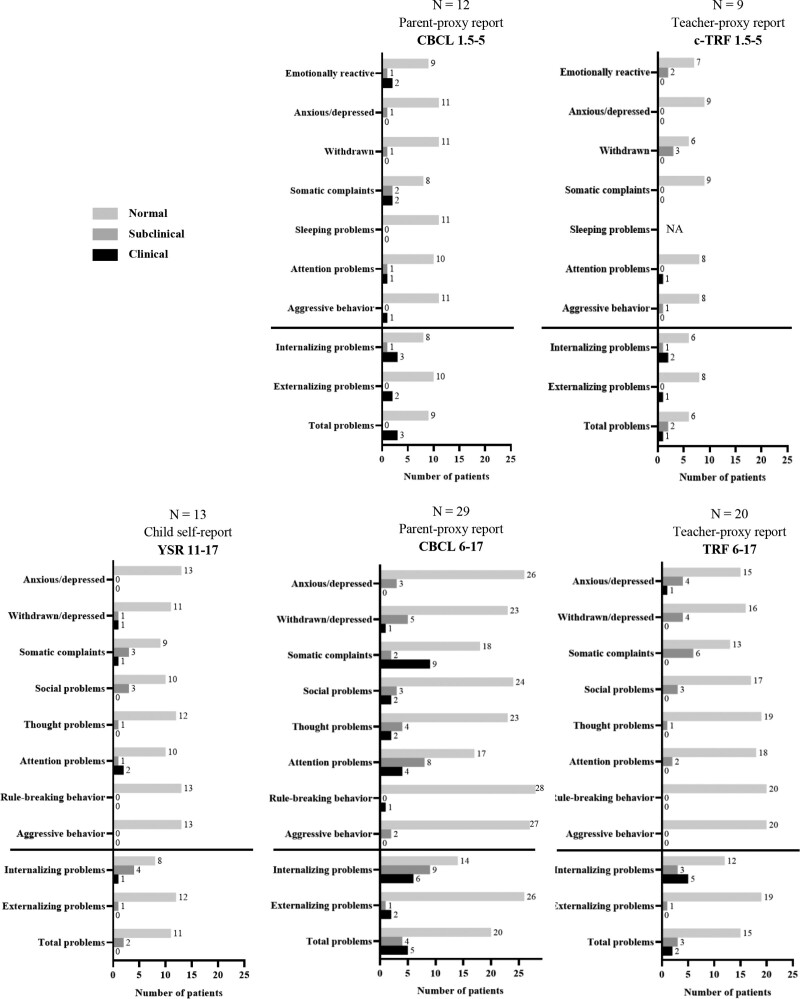
Severity of problems in children reported by children (YSR), parents (CBCL), and teachers (TRF). CBCL = Child Behavior Checklist (1.5–5 and 6–17 years); NA = not applicable; TRF = Teacher’s Report Form (1.5–5 and 6–17 years); YSR = Youth Self Report (11–18 years). For syndrome scale scores: T-score ≤63: normal problems, T-score 64–69: subclinical problems, T-score ≥70: clinical problems. For internalizing/externalizing/total problem scores: T-score ≤59: normal problems, T-score 60–63: subclinical problem, T-score ≥64: clinical problems.

Compared with normative data, children aged ≥11 years (13 of 14 eligible children) reported significantly more internalizing problems (Table 2). Subclinical or clinical internalizing problems were reported by 5 children (38%), which is significantly more than in the general population (17%, *P* < 0.001; Fig. 1).

**TABLE 2.** Emotional (internalizing) and behavioral (externalizing) problems

Scale	T-score YSR self-report	*P* value*	Cohen *d*	T-score CBCL parent-proxy	*P* value*	Cohen *d*	T-score (c-)TRF teacher-proxy	*P* value*	Cohen *d*
	Median [IQR]			Median [IQR]			Median [IQR]		
Children aged 1.5–5 y	–			N = 12			N = 9		
*Syndrome scales*									
Internalizing									
Emotionally reactive	NA	NA	NA	59.0 [50.0–67.0]	**0.017**		51.0 [50.0–63.0]	**0.043**	
Anxious/depressed	NA	NA	NA	50.5 [50.0–60.3]	**0.027**		54.0 [50.5–59.0]	**0.017**	
Withdrawn	NA	NA	NA	51.0 [51.0–62.3]	**0.008**		56.0 [51.5–66.0]	**0.012**	
Somatic complaints	NA	NA	NA	60.0 [50.8–67.3]	**0.003**		50.0 [50.0–57.0]	0.059	
Sleeping problems	NA	NA	NA	50.5 [50.0–56.0]	**0.027**		NA		
Externalizing									
Attention problems	NA	NA	NA	53.0 [51.5–57.0]	**0.003**		55.0 [51.0–60.5]	**0.018**	
Aggressive behavior	NA	NA	NA	50.0 [50.0–53.8]	0.109		53.0 [50.0–56.0]	**0.042**	
*Aggregate scales*									
Internalizing problems	NA	NA	NA	53.0 [43.5–67.0]	0.201	0.4	52.0 [46.5–63.0]	0.212	0.5
Externalizing problems	NA	NA	NA	48.5 [42.3–53.8]	0.825	0.1	52.0 [48.0–57.0]	0.257	0.4
Total problems	NA	NA	NA	49.5 [41.8–63.0]	0.464	0.2	54.0 [48.0–60.0]	0.135	0.6
Children aged 6–17 y	N = 13			N = 29			N = 20		
*Syndrome scales*									
Internalizing									
Anxious/depressed	52.0 [50.0–56.5]	**0.008**		52.0 [51.0–59.0]	**<0.001**		57.0 [50.3–63.5]	**<0.001**	
Withdrawn/depressed	54.0 [50.0–60.5]	**0.001**		56.5 [52.5–61.5]	**<0.001**		53.0 [50.0–61.8]	**0.001**	
Somatic complaints	60.0 [52.0–65.5]	**0.007**		59.5 [51.5–70.0]	**<0.001**		61.0 [50.0–65.0]	**<0.001**	
Social problems	57.0 [51.0–63.5]	**0.005**		53.5 [51.3–61.8]	**<0.001**		55.5 [50.0–61.3]	**0.001**	
Thought problems	51.0 [50.0–56.5]	**0.018**		53.0 [50.0–63.0]	**<0.001**		50.0 [50.0–57.0]	**0.014**	
Attention problems	53.0 [50.0–64.5]	**0.008**		61.0 [51.3–66.5]	**<0.001**		52.0 [50.0–58.8]	**0.003**	
Externalizing									
Rule-breaking behavior	51.0 [50.0–51.5]	**0.010**		51.0 [50.0–54.8]	**<0.001**		50.0 [50.0–53.0]	**0.027**	
Aggressive behavior	50.0 [50.0–52.0]	0.068		51.0 [50.0–56.8]	**<0.001**		53.0 [50.0–55.0]	**0.003**	
*Aggregate scales*									
Internalizing problems	56.0 [52.0–61.5]	**0.011**	0.8	60.0 [49.8–62.8]	**<0.001**	0.9	57.5 [49.0–63.8]	**0.004**	0.7
Externalizing problems	44.0 [40.0–49.0]	**0.036**	−0.7	48.5 [41.8–56.0]	0.546	−0.1	51.0 [43.0–54.8]	0.506	−0.2
Total problems	53.0 [45.0–56.5]	0.688	0.1	56.0 [49.0–62.0]	**0.002**	0.6	53.5 [48.3–60.3]	**0.017**	0.6

*P* values in bold are significantly different from normative data. Example: children reported significantly more internalizing/emotional problems (higher score: 56 vs 50, *P* = 0.011) than their peers, whereas they reported significantly less externalizing/behavioral problems (lower score: 44 vs 50, *P* = 0.036) than their peers.

CBCL = Child Behavior Checklist; c-TRF = caregiver Teacher’s Report Form; NA = not applicable; TRF = Teacher’s Report Form; YSR = Youth Self Report (11–18 years).

* Compared with normative data (T-score = 50, SD = 10). Tested with one-sample Wilcoxon signed rank tests and 1-sample *t* tests (depending on normality of the data distribution).

##### CBCL (Parent-Reported Problems)

In preschool children [1.5–5 years, n = 12 (all eligible children)], parents reported similar scores on total problems, internalizing problems, and externalizing problems compared with normative data. In school-aged children [6–17 years, n = 29 (all eligible children)], compared with normative data, parents reported significantly higher scores on total and internalizing problems (Table 2). For all children together (n = 41), subclinical or clinical internalizing problems in children were reported by 19 parents (46% vs 17%, *P* < 0.001; Fig. 1).

##### TRF (Teacher-Reported Problems)

In preschool children [1.5–5 years, n = 9 (of 12 eligible children)], teachers reported similar scores on total problems, internalizing problems, and externalizing problems compared with normative data. In school-aged children [6–17 years, n = 20 (of 29 eligible children)], teachers reported significantly higher scores on total and internalizing problems compared with normative data (Table 2). For all children together [n = 29 (of 41 eligible children; in 12 children, no teacher filled out the questionnaire)], subclinical or clinical internalizing problems were reported by 11 teachers (38% vs 17%, *P* = 0.006; Fig. 1).

### Medical Traumatic Stress

Nineteen children (8–17 years, of 23 eligible children) filled out the CRTI, of whom 1 (5%) had elevated post-traumatic stress. Their mean *z* score was −1.2 (SD 0.8), which was significantly lower than normative data (*z* score = 0, *P* < 0.001), indicating less medical traumatic stress. Thirty-seven parents (4–17 years, of 41 eligible parents) also reported significantly less medical traumatic stress compared to norm data [*z* score of −0.7 (SD 0.8)]. For five children (14%), parents reported elevated post-traumatic stress. Nine children (22%) underwent Eye Movement Desensitization and Reprocessing (EMDR) therapy for trauma-related problems prior to or during the current study.

### Children Receiving PN Versus Children Weaned off PN

When comparing children still receiving PN with children weaned off PN, no significant differences in total, internalizing, or externalizing problems T-scores were seen (in reports from either children themselves, parents, or teachers for all ages). Also, for medical traumatic stress, no differences were seen (Table 1, Supplemental Digital Content 3, http://links.lww.com/MPG/D307).

### Risk Factors for Adverse Outcomes

Lower GI-related quality of life was significantly associated with more parent-reported internalizing problems when correcting for gestational age and child’s age at assessment. Both lower age at time of assessment and lower GI-related quality of life were significant predictors of more parent-reported externalizing problems. There were no significant associations between clinical variables and parent-reported traumatic problems. However, for self-reported traumatic problems, number of surgeries (*β* = −0.5, *P* = 0.031) and PN-dependency duration (*β* = −0.6, *P* = 0.012) were inversely associated with traumatic problems, indicating that more surgeries and longer PN-duration were associated with less medical traumatic stress. In Table 3, univariable and multivariable analyses for predictors of internalizing problems, externalizing problems, and traumatic problems are shown.

**TABLE 3.** Associations of clinical characteristics with *internalizing* problems, *externalizing* problems, and *traumatic* problems (univariable and multivariable linear regression analyses)

	Univariable analysis					Multivariable analysis				
	*B*	SE	95% CI	*β*	*P* value	*B*	SE	95% CI	*β*	*P* value
		Parent-proxy report internalizing problems					Parent-proxy report *internalizing* problems			
		N = 41					N = 41			
Sex, female	1.57	3.28	−5.07 – 8.21	0.08	0.635					
Age at time of assessment, y	0.73	0.38	−0.05 – 1.50	0.30	0.064	0.14	0.30	−0.46 – 0.74	0.06	0.636
Gestational age, wk	−0.02	0.37	−0.77 – 0.74	−0.01	0.964					
Duration of hospital stays, mo	−0.12	0.25	−0.63 – 0.40	−0.27	0.651					
Number of surgeries	−0.08	0.25	−0.59 – 0.43	−0.05	0.762					
Duration of PN-dependency, mo	0.01	0.03	−0.06 – 0.08	0.05	0.782					
PedsQL GI total score	−6.14	0.95	−8.07 – 4.22	−0.72	**<0.001**	−5.98	1.02	−8.05 – −3.91	−0.70	**<0.001**
		Parent-proxy report *externalizing* problems					Parent-proxy report *externalizing* problems			
	N = 41							N = 41		
Sex, female	−1.05	3.51	−8.15 – 6.06	−0.05	0.767					
Age at time of assessment, y	−0.71	0.41	−1.54 – 0.12	−0.27	0.090	−1.09	0.44	−1.98 – −0.20	−0.40	**0.018**
Gestational age, wk	−0.60	0.38	−1.38 – 0.17	−0.25	0.123	−0.46	0.36	−1.19 – 0.28	−0.19	0.215
Duration of hospital stays, mo	−0.20	0.27	−0.74 – 0.35	−0.12	0.467					
Number of surgeries	−0.33	0.26	−0.86 – 0.21	−0.20	0.224					
Duration of PN-dependency, mo	−0.01	0.04	−0.08 – 0.07	−0.02	0.885					
PedsQL GI total score	−0.22	0.14	−0.51 – 0.07	−0.25	0.125	−0.37	0.14	−0.65 – −0.08	−0.41	**0.013**
		Parent-proxy report *traumatic* problems					Parent-proxy report *traumatic* problems			
			N = 37					N = 37		
Sex, female	0.27	0.28	−0.29 – 0.83	0.16	0.340	0.20	0.27	−0.35 – 0.75	0.12	0.471
Age at time of assessment, y	0.01	0.04	−0.06 – 0.09	0.05	0.773					
Gestational age, wk	0.02	0.03	−0.04 – 0.08	0.14	0.423					
Duration of hospital stays, mo	−0.01	0.02	−0.06 – 0.04	−0.07	0.689					
Number of surgeries	−0.01	0.02	−0.05 – 0.04	−0.01	0.942					
Duration of PN-dependency, mo	−0.01	0.01	−0.01 – 0.01	−0.03	0.865					
PedsQL GI total score	−0.20	0.11	−0.42 – 0.02	−0.30	0.075	−0.19	0.11	−0.42 – 0.04	−0.28	0.100

*P* values in bold represent significant associations. Example interpretation of multivariable linear analysis: when age remains the same and the parent-reported PedsQL GI total score increases with 10, its parent-reported T-score on internalizing problems in their child decreases with ~6 (5.98) points (ie, better quality of life concerning gastrointestinal complaints and worries is associated with less internalizing problems). 95% CI = 95% confidence interval of unstandardized coefficient; β = standardized coefficient; *B* = unstandardized coefficient; PedsQL GI = Pediatric Quality of Life inventory – Gastrointestinal Symptoms Module; PN = parenteral nutrition; SE = standard error of unstandardized coefficient.

Medical traumatic stress was associated with emotional problems (*β* = 0.3, *P* = 0.042).

## DISCUSSION

In this exploratory 2-center study, we found that psychopathology was common in children with IF and that more than one-fifth of the children had already received psychological treatment for medical traumatic stress problems. Lower GI-related quality of life was associated with both emotional and behavioral problems.

### Psychopathology

#### Psychiatric Classifications

The number of psychiatric classifications is relatively high in the IF-population. There are no Dutch normative data available for the MINI-KID, but there are Dutch population-based studies assessing DSM-IV classifications using comparable tools. Scholte et al (27) reported 14% of children from the general population (4–17 years) fulfilling criteria of 1 or more current DSM-IV psychiatric classifications, compared with 33% of our cohort. Ormel et al (28) found lifetime prevalences in children from the general population (11–17 years) of 12% with anxiety disorders (both specific phobia and social phobia) and 4% with ADHD, compared with 19% anxiety disorders and 11% ADHD in our cohort.

#### Emotional and Behavioral Problems

Disturbingly and in line with psychiatric outcomes, subclinical or clinical emotional problems were reported in almost half of the children. This is in line with findings of previous studies concerning children with IF. Already in 1989, in a cohort of 16 children (2–12 years) with short bowel syndrome, increased emotional and behavioral difficulties were found including anxiety, infantile behavior, attention problems, and poor school results (5). In another study, involving 25 children receiving home PN (3–15 years), problems with activities and social interaction were observed. Parents considered their children to be anxious, shy, and sensitive. Similar to our cohort, behavioral problems were not over represented (6). In a recent study from Bondi et al (9) including a Canadian IF-population, poorer emotional functioning was reported too, indicating this is an internationally seen phenomenon which underscores the urgency to act. Increased behavioral difficulties were reported in their cohort, which in our cohort was not confirmed. Children from our cohort were slightly older (mean age 8.9 vs 6.6 years) and less often male (45% vs 55%). Since behavioral problems are generally seen more often in younger children and boys, this may explain the difference (29). One may think that the reported increased emotional problems can be fully explained by increased somatic problems, but this is not the case, since on all syndrome scales of emotional/internalizing problems (ie, emotionally reactive, anxious/depressed, withdrawn and somatic complaints) increased problems were reported. Despite advancements in treatment and monitoring by multidisciplinary IF teams over the years, mental health problems are still high in children with IF, especially when bearing in mind that almost one-third had already received psychological help before taking part in the study. Prevalence of emotional problems varies in children with other GI or chronic diseases such as inflammatory bowel disease (18%) (30), gastroschisis (33%) (31), dilated cardiomyopathy (39%) (32), and cystic fibrosis (16%) (33), or children born preterm (10%) (34), all reporting lower prevalences compared with children with IF (46%), except for children with anorectal malformations (59%) (35). Looking at it from a more positive perspective, more than half of the children does not experience emotional problems (anymore) after a period of critical illness at the start of their lives.

An explanation for the psychopathology found in children with IF involving the gut-brain axis might be found in alterations of the gut microbiome. Previous studies showed that a decrease in short-chain fatty acid producing species and an increase in inflammatory species are associated with anxiety disorders (36,37) and ADHD (38). These are also characteristics of the gut microbiome profile found in children with IF (39,40). A higher prevalence of both anxiety disorders and ADHD was seen in other pediatric GI diseases such as inflammatory bowel disease (41) and coeliac disease as well, suggesting involvement of the gut-brain axis (42).

#### Risk Factors for Adverse Outcomes

The increased emotional problems are not likely to be explained by PN-related factors, since we found no association between PN-dependency duration and emotional problems. This is in line with our finding that emotional problem scores of patients weaned off PN did not differ from patients still receiving PN. We expected longer PN-dependency duration to be associated with worse emotional and behavioral functioning, because having a central venous line may impair children in their daily functioning because of limited freedom of movement and a different experience of social mealtimes and activities. Possibly, children with longer PN-dependency have adapted to the situation. Moreover, events in early life such as multiple surgeries and long hospitalization in a period of critical illness and associated disturbed parent–child relationship may affect emotional functioning (43), rather than current PN-dependency. We did find an association between lower GI-related quality of life and more emotional problems, again emphasizing the bidirectional communication between the central and the enteric nervous system in the gut-brain axis (44). Of note is that more children still receiving PN than children weaned off PN received psychological treatment. Possibly, their need for psychological support is more often acknowledged, but this was not reflected by a lower rate of psychopathology.

Interestingly, reported emotional problem scores in preschool children (1.5–5 years) were not higher than normative data scores. This may be because this group was too small to find significant differences. However, subclinical or clinical emotional problems were reported by parents and teachers in 33% of preschool children, which remains high and warrants attention from clinicians.

### Medical Traumatic Stress

Surprisingly, not many children with IF seemed to suffer from traumatic complaints following multiple hospital visits and surgeries. In a study including children who had been hospitalized for minimally 1 night in a Dutch hospital (for varying medical reasons/etiologies), medical traumatic stress was assessed using the same questionnaire we used (23). In that study, elevated medically related post-traumatic stress was found in 22% of self-reports and 21% of parent-proxy reports, compared with 5% and 14%, respectively, in our cohort. In a study including children with inflammatory bowel disease, pancreatitis, and cystic fibrosis, medical traumatic stress was assessed with a different questionnaire. Medical traumatic stress was present in 36% of the children and was associated with medication burden, emergency and intensive care visits, and parent post-traumatic stress disorder, emphasizing the need for support in these chronic pediatric populations (45). We did not systematically investigate the type of therapy, but the parents of 22% of the children told us that their child had received EMDR therapy for traumatic complaints. This may have helped in dealing with medical traumatic stress because EMDR is proven to be an effective therapy for traumatic complaints (46). Surprisingly, more surgeries and longer PN-duration were associated with less traumatic stress according to self-reports. Possibly, these children have learned to cope with their situation over time, since children with chronic conditions are often found to be resilient (47,48). Given the early age of diagnosis and hospital admissions during infancy, there may be preverbal trauma present in these children which was not adequately addressed in the questionnaire we used.

### Strengths and Limitations

To the best of our knowledge, this is the first study assessing the presence of psychiatric classifications and medical traumatic stress in children with IF. It was 2-center including multiple methods to assess psychological problems. Another strength is that we also included children who already weaned off PN. Inevitably, there are some limitations that need to be considered when interpreting the findings. This study was performed partly during COVID-19 pandemic which could have affected the results. From a Dutch study in the general pediatric population, we know that significantly more emotional problems were seen during the first peak of the pandemic compared to pre-pandemic (49). Yet, during the course of the pandemic problems did not differ, were lower, or subsided. Children were mostly concerned about home confinement (49,50). When the study was performed, children were allowed to go to daycare and school again. Another limitation of the current study is that one of the caregivers was present during the MINI-KID interview, possibly influencing the child’s answers to questions. Including children in the study who already received psychological help before, may have resulted in an underestimation of for example the number of children reporting traumatic complaints due to positive response to previous treatment. However, excluding them, would not give a true view of the population and the psychological problems they have faced. The sample size was small, which is inherent to the rarity of IF. We tried to increase the sample size by adding another center to the study. However, the participation rate of approximately 49% in Rotterdam and 15% in Amsterdam (overall 37%) might have led to participation bias. Other pediatric studies using comparable questionnaires had response rates of 49%, 46%, and 34% (51,52). For the children from Amsterdam, the travel time to Rotterdam was the greatest barrier. In general, nonparticipants did not want to take part because of not wanting to go to hospital during the pandemic, lack of time due to being overloaded with caring for their child, or their child already being evaluated for psychological problems elsewhere. The latter 2 underscore the demand for psychological support for families with children with IF. On the other hand, it may also be possible that parents of children struggling with mental health were more likely to participate in this study (although this was not an intervention study).

### Implications for Clinical Practice and Future Research

Our results highlight the need for mental health monitoring and support as an integrated part of the management of pediatric patients with IF. From 2 large international surveys on the composition of multidisciplinary IF teams, we know that in Latin America, approximately 58% has a psychologist in the team (53) and in Europe, this is 66% (54). Based on the findings of the current study, we believe this percentage should be 100%. Currently, psychological treatment is mostly reactive when problems have emerged or escalated. With early screening and detection of problems, we can offer timely treatment to prevent psychopathology and trauma later in life. Interventions should be both prevention and treatment initiatives for all children with IF and their families. This could be in the form of a family centered approach (55), cognitive behavioral therapy (56), or an exposure based treatment such as EMDR or integrative psychotherapy (57), targeting both medical traumatic stress and emotional stress, since these were associated with each other. Future studies should focus on the effectiveness and timing of these prevention and treatment strategies in this vulnerable pediatric population.

## CONCLUSIONS

Children with IF are at risk for psychopathology and also show heightened rate of medical traumatic stress. These adverse outcomes are also common in children already weaned off PN and are associated with the presence of GI-related symptoms, rather than PN-dependency duration or prematurity. Our findings highlight the need for timely psychological screening and support for children with IF and their families.

## Acknowledgments

We are grateful to all the children and their parents who took part in the current study. We thank everyone who contributed to the crowd funding of the “Sporten voor Sophia” event, which made it possible to do research on cognitive and psychosocial outcomes in children with IF.

## Supplementary Material


