# Supplementary material for: Children with Intestinal Failure are at Risk for Psychopathology and Trauma
Source: J Pediatr Gastroenterol Nutr. 2023 Sep 8;77(6):e104–13. doi: 10.1097/MPG.0000000000003939 (PMC10642705; doi:10.1097/MPG.0000000000003939)
Supplement: Supplementary file 1 [file mpg-77-e104-s001.pdf]

File S1

Detailed description of the used interview and questionnaires

Psychopathology

Interview: psychiatric classifications

Psychiatric classifications in the child were assessed with the translated and linguistically validated Dutch version of the Mini International Neuropsychiatric Interview Kid Screen (MINI-KID). The MINI-KID is a semi-structured interview, carried out by trained personnel, used for determining psychiatric classifications (Diagnostic and Statistical Manual of Mental Disorders (DSM-) IV-TR diagnoses) in children and adolescents aged 6 to 18 years.[1] The MINI-KID generates reliable and valid psychiatric classifications.[2] The MINI-KID includes modules covering depressive disorders, suicidality, bipolar disorders, anxiety disorders, obsessive compulsive disorders, posttraumatic stress disorders, alcohol abuse, substance abuse, tic disorders, attention deficit disorder with hyperactivity (ADHD), disruptive disorders, psychotic disorders, eating disorders and autism spectrum disorders. Both child/adolescent and the primary caregiver were present during the interview with the psychologist. The primary caregiver was present for emotional support and in general not answering questions. For the younger children, some questions appeared to be too difficult. Only in the case that the child did not know what to answer or did not understand the question, his/her primary caregiver would answer on behalf of them.

Questionnaire: emotional and behavioral problems

Emotional and behavioral functioning in children was assessed with the Dutch versions of the Youth Self Report (YSR, for 11-18 years), the Child Behavior Checklist (CBCL for 1.5-5 years

and 6-18 years) as parent proxy-report and the Teacher's Report Form (TRF, for 1.5-5 years and 6-18 years) as daycare staff/teacher proxy-report. The YSR, CBCL and TRF have shown good reliability and validity.[3] The YSR, CBCL and TRF are questionnaires used for quantifying internalizing problems (emotional functioning, i.e. problems that are present mainly within the child), externalizing problems (behavioral functioning, i.e. problems with regard to conflicts with others and to conflicts in the expectation that others have of the child) and total problems (i.e. internalizing and externalizing problems together). The answers to the questions are rated on a 3-point Likert scale resulting in a total raw score, which is transformed into an age and sex corrected T-score.[4 5 5, 6]

Higher scores implicate more psychopathology; with T-scores of 60-64 reflecting subclinical/borderline problems (i.e., at risk of becoming clinical problems) and T-scores of 65 and higher representing clinical problems (i.e., requiring treatment). In the general population, 9% (84th – 92nd percentile) is known to have borderline problems, and 8% (93rd – 100th percentile) is known to have clinical problems.[5] In addition to the composite T-scores, problems can be categorized in syndrome scales, namely: emotionally reactive, anxious/depressed, withdrawn/depressed, somatic complaints, sleeping problems, social problems, attention problems, thought problems, rule-breaking behavior, and aggressive behavior.

Trauma

Medical traumatic stress in the children was assessed with the validated Dutch Children's Responses to Trauma Inventory (CRTI).[7, 8] It has a child self-report (8-18 years) and a parent-proxy report (4-18 years). The CRTI assesses how a child feels regarding a traumatic event. Parents and children were instructed to think about hospital visits, admissions and surgeries when filling

out the questionnaire. Items are rated on a 5-point Likert scale, leading to a total score, based on four subscales: intrusion, avoidance, arousal and other child-specific reactions. Higher scores indicate more medical traumatic stress. The post-traumatic stress disorder (PTSD) total score was computed with the 24 PTSD-items. A child with a PTSD total score above the 60th percentile and/or fulfilling two or three of the three symptom criteria was categorized as having 'elevated PTSS'. Sex and age adjusted Z-scores can be calculated based on normative data.[7, 9]

Gastrointestinal related quality of life

Feeding difficulties and gastrointestinal complaints were assessed with the Pediatric Quality of Life inventory – Gastrointestinal Symptoms Module (PedsQL GI). This is a questionnaire filled out by children themselves (≥5 years, self-report) and their parents (2-18 years, proxy-report).[10] The total score is based on several symptom subscale scores, such as abdominal pain, trouble swallowing, food and drink limits, nausea and vomiting, and diarrhea, together with a score for worries about complaints, medication and talking about their illness to others. The total score can range between 0 and 100 with higher scores indicating better quality of life with less GI-related complaints and worries. The PedsQL GI has been validated in patients with several gastrointestinal conditions (such as Crohn's disease, gastroesophageal reflux disease and functional gastrointestinal disorders).[11]

REFERENCES

[1] Bauhuis O, Jonker K, Verdellen C, Reynders J, Verbraak M. De introductie van een Nederlandstalig instrument om DSM-IV-Tr-diagnoses bij kinderen te stellen. Kind & Adolescent Praktijk. 2013;12:20-6.
[2] Sheehan DV, Sheehan KH, Shytle RD, Janavs J, Bannon Y, Rogers JE, et al. Reliability and validity of the mini international neuropsychiatric interview for children and adolescents (MINI-KID). The Journal of clinical psychiatry. 2010.
[3] Holmbeck GN, Thill AW, Bachanas P, Garber J, Miller KB, Abad M, et al. Evidence-based assessment in pediatric psychology: Measures of psychosocial adjustment and psychopathology. Journal of Pediatric Psychology. 2008;33:958-80.
[4] Achenbach TM, Rescorla LA. Manual ASEBA Child Behavior Checklists for Ages 1.5–5 Years (CBCL/1.5–5). ASEBA, Vermont University: Burlington, VT, USA. 2001.
[5] Achenbach TM, Rescorla LA. Manual for the ASEBA school-age forms & profiles: child behavior checklist for ages 6-18, teacher's report form, youth self-report: an integrated system of multi-informant assessment: University of Vermont, research center for children youth & families; 2001.
[6] Achenbach TM, Rescorla LA. Manual for the ASEBA preschool forms and profiles: Burlington, VT: University of Vermont, Research center for children, youth …; 2000.
[7] Alisic E, Eland J, Kleber RJ. Schokverwerkingslijst voor Kinderen-herziene versie [Children's responses to trauma inventory-revised version]. Zaltbommel/Utrecht, the Netherlands: Institute for Psychotrauma in collaboration with Clinical Psychology Utrecht University and Psychotrauma Center for Children and Youth (UMC Utrecht). 2006.
[8] Alisic E, Kleber RJ. Measuring Posttraumatic Stress Reactions in Children: A Preliminary Validation of the Children's Responses to Trauma Inventory. Journal of Child & Adolescent Trauma. 2010;3:192-204.
[9] Meentken MG, van der Ende J, del Canho R, van Beynum IM, Aendekerk EWC, Legerstee JS, et al. Psychological outcomes after pediatric hospitalization: the role of trauma type. Children's Health Care. 2021;50:278-92.
[10] Varni JW, Kay MT, Limbers CA, Franciosi JP, Pohl JF. PedsQL gastrointestinal symptoms module item development: qualitative methods. J Pediatr Gastroenterol Nutr. 2012;54:664-71.
[11] Varni JW, Bendo CB, Shulman RJ, Self MM, Nurko S, Franciosi JP, et al. Interpretability of the PedsQL Gastrointestinal Symptoms Scales and Gastrointestinal Worry Scales in Pediatric Patients With Functional and Organic Gastrointestinal Diseases. J Pediatr Psychol. 2015;40:591-601.
