# Supplementary material for: Children with Intestinal Failure are at Risk for Psychopathology and Trauma
Source: J Pediatr Gastroenterol Nutr. 2023 Sep 8;77(6):e104–13. doi: 10.1097/MPG.0000000000003939 (PMC10642705; doi:10.1097/MPG.0000000000003939)
Supplement: Supplementary file 2 [file mpg-77-e104-s002.pdf]

**File S2**

***A detailed description of the statistical analysis***

## Statistical analysis

Values are expressed as median with 25th to 75th percentile (interquartile range: IQR) (for continuous data), or as an amount (n) with percentage (%) (for categorical data). The prevalence of psychiatric classifications is described, but not statistically compared since there are no normative data available. Chi-square tests, Mann Whitney U tests and independent t-tests were used to compare characteristics and emotional and behavioral functioning between the group of children still receiving PN and the group of children weaned off of PN. These groups were compared to evaluate whether PN itself or other more generic disease factors influenced psychosocial functioning and if problems resolve once weaned off PN. One-sample t-tests and one-sample Wilcoxon signed rank tests were used to compare total, internalizing and externalizing T-scores with normative data (general population mean T-score = 50, SD = 10) and we also calculated Cohen's *d* effect sizes (with Cohen's *d* <0.5 considered a small effect, Cohen's *d* 0.50-0.79 a medium effect and Cohen's *d* ≥0.8 a large effect) (1). Binomial tests were performed to compare the proportion of children with borderline or clinical problems with the general population (17%) (2, 3). Furthermore, one-sample Wilcoxon signed rank tests were used to compare traumatic complaints Z-scores with the population norm (Z-score = 0).

We used PN-dependency duration and gastrointestinal (GI) related quality of life (measured with the Pediatric Quality of Life inventory – Gastrointestinal Symptoms Module (PedsQL GI) total score) as proxies of IF severity. We performed explorative univariable linear regression analyses to assess associations of clinical characteristics (such as PN-dependency duration and GI-related

quality of life) with internalizing and externalizing problems, and also with medical traumatic stress. This was done for the parent-proxy reports, since these encompass all children. Due to the sample size and variety of underlying diseases, we were not able to include IF diagnosis/underlying disease as a predictor. Predictors with $p < 0.4$ were then combined in multivariable linear regression analyses with a maximum of one predictor added for every n = 10 children.

A two-tailed *p*-value of <0.05 was considered to indicate statistical significance. Data analyses were performed using the Statistical Package for the Social Sciences, Version 25.0 (IBM SPSS Statistics for Windows, Armonk, NY, USA).

## REFERENCES

1. Cohen J. Statistical power analysis for the behavioral sciences. Lawrence Erlbaum Associates. Hillsdale, NJ. 1988:20-6.
2. Achenbach TM, Rescorla LA. Manual for the ASEBA school-age forms & profiles: child behavior checklist for ages 6-18, teacher's report form, youth self-report: an integrated system of multi-informant assessment: University of Vermont, research center for children youth & families; 2001.
3. Achenbach TM, Rescorla LA. Manual for the ASEBA preschool forms and profiles: Burlington, VT: University of Vermont, Research center for children, youth …; 2000.
