# Supplementary material for: Children with Intestinal Failure are at Risk for Psychopathology and Trauma
Source: J Pediatr Gastroenterol Nutr. 2023 Sep 8;77(6):e104–13. doi: 10.1097/MPG.0000000000003939 (PMC10642705; doi:10.1097/MPG.0000000000003939)
Supplement: Supplementary file 3 [file mpg-77-e104-s003.pdf]

Table S1. Comparison of problems between children weaned off PN and receiving PN

		**Weaned off PN at time of assessment**<br>**N = 24**<br>Median [IQR]	**Receiving PN at time of assessment**<br>**N = 17**<br>Median [IQR]	***p*-value**
**YSR T-score (11-17y)**	n = 13			
Internalizing problems		62.0 [49.0 – 64.0]	55.0 [51.5 – 59.0]	0.284
Externalizing problems		46.0 [39.5 – 49.0]	44.0 [40.0 – 53.8]	0.943
Total problems		54.0 [43.5 – 59.5]	50.5 [46.0 – 55.8]	0.833
**CBCL T-score (4-17y)**	n = 41			
Internalizing problems		60.0 [45.0 – 67.0]	58.0 [54.0 – 61.5]	0.745
Externalizing problems		47.0 [40.0 – 56.0]	49.0 [44.0 – 55.5]	0.551
Total problems		51.0 [44.0 – 64.0]	56.0 [52.0 – 59.0]	0.356
**TRF T-score (4-17y)**	n = 29			
Internalizing problems		55.0 [47.5 – 66.0]	55.0 [47.3 – 62.5]	0.744
Externalizing problems		52.0 [43.0 – 55.0]	50.0 [42.3 – 53.8]	0.471
Total problems		55.0 [48.5 – 62.0]	52.0 [48.3 – 55.8]	0.283
**CRTI child self-report Z-score (8-17y)**	n = 19			
Traumatic problems		-1.1 [-1.6 – -0.5]	-1.4 [-2.2 – -0.8]	0.422
**CRTI parent-proxy report Z-score (4-17y)**	n = 37			
Traumatic problems		-0.8 [-1.3 – 0.1]	-0.7 [-1.4 – -0.4]	0.511

CBCL: Child Behavior Checklist, CRTI: Children’s Responses to Trauma Inventory, PN: parenteral nutrition, TRF: Teacher’s Report Form, YSR: Youth Self Report.
